# Overview of Monogenic Forms of Hypertension Combined With Hypokalemia

**DOI:** 10.3389/fped.2020.543309

**Published:** 2021-01-25

**Authors:** Yi-Ting Lu, Peng Fan, Di Zhang, Ying Zhang, Xu Meng, Qiong-Yu Zhang, Lin Zhao, Kun-Qi Yang, Xian-Liang Zhou

**Affiliations:** Department of Cardiology, Fuwai Hospital, National Center for Cardiovascular Diseases, Chinese Academy of Medical Sciences and Peking Union Medical College, Beijing, China

**Keywords:** monogenic hypertension, hypokalemia, phenotypic variability, pathophysiology, genetic sequencing

## Abstract

Hypertension is an important risk factor in many conditions and creates a heavy burden of disease and mortality globally. Polygenic hypertension is the most common form; however, it is increasingly recognized that monogenic hypertension is not rare, especially in patients with electrolyte disorders. Single genetic alterations are associated with plasma volume expansion and catecholamines/sympathetic excess with simultaneously increased potassium excretion in the urine and potassium intracellular shift. Early-onset refractory hypertension and profound hypokalemia are characteristics of monogenic hypertension. However, accumulated evidence shows the existence of phenotypic heterogeneity in monogenic hypertension meaning that, even for mild symptoms, clinicians cannot easily exclude the possibility of monogenic hypertension. Genetic, epigenetic and non-genetic factors are all possible mechanisms influencing phenotypic diversity. Genetic sequencing is a precise and efficient method that can broaden the mutant gene spectrum of the disease and is very helpful for understanding the pathophysiology of monogenic hypertension. Genetic sequencing, along with biochemical tests and imaging modalities, is essential for the early diagnosis and targeted management of monogenic hypertension to avoid long-term catastrophic complications.

## Introduction

Hypertension is one of the leading causes of death worldwide, affecting more than 1.1 billion people (1), and is a major risk factor for cardiovascular disease (CVD) and stroke (2). On the basis of its genetic contribution, hypertension can be classified into two types: polygenic hypertension and monogenic hypertension. Polygenic hypertension is affected by complex genetic variants and also lifestyle and environmental factors, such as smoking status, alcohol consumption, dietary salt intake, obesity, and physical motion (3, 4). Polygenic hypertension without identifiable causes is the most common form. In contrast, monogenic hypertension is an inherited hypertension disease caused by single genetic variants that follow Mendelian inheritance.

**Figure 1:**
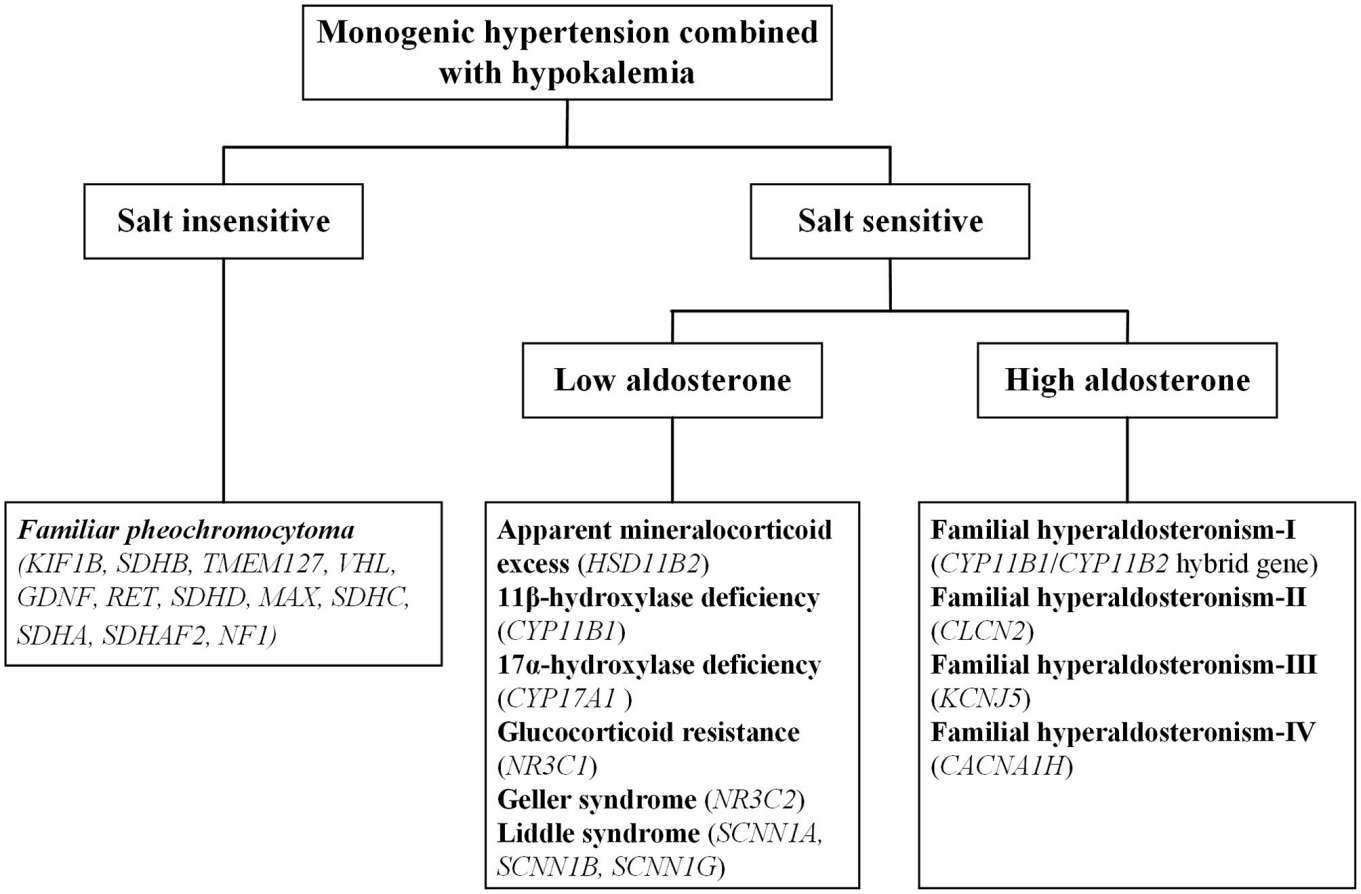
The overall categorization of monogenic hypertension combined with hypokalemia.

Monogenic hypertension is almost always associated with electrolyte disturbances, with hypokalemia commonly seen. Based on salt sensitivity and the level of aldosterone, monogenic hypertension combined with hypokalemia could be classified into three categories, salt insensitive hypertension, salt-sensitive hypertension with low/high aldosterone levels (Figure 1). Hypokalemia is generally attributed to increased potassium excretion or intracellular metastasis, while decreased potassium intake is relatively uncommon (5). Low potassium levels can induce multiple cardiac blocks or arrhythmias, sometimes requiring emergency medical care (6). Refractory hypertension is also frequent in patients with monogenic hypertension, defined as the failure to control blood pressure when given five or more various classes of antihypertensive agents (7). Long-term uncontrolled hypertension may develop complex complications, including severe targeted organ damage and even cardiovascular death events. Early recognition and individualized treatment are of paramount importance for the above situations.

There are three basic mechanisms leading to the condition of expansive volume and hypokalemia: mineralocorticoid excess, salt retention and sympathetic activation. On this basis, various forms of monogenic hypokalemic hypertension commonly manifest as early-onset refractory hypertension, profound hypokalemia and metabolic alkalosis; except for pheochromocytoma (PCC), all forms of monogenic hypertension also have low levels of renin. However, it is vital to note that not all monogenic hypertension patients present with typical phenotypes. In recent years, advanced gene testing techniques have identified a series of atypical cases with monogenic hypertension that may previously have been overlooked or misdiagnosed as primary hypertension (8–11).

In this review, we summarize different kinds of monogenic hypertension combined with hypokalemia. We describe their mechanisms, clinical manifestations, and treatment strategies. Additionally, possible reasons for the phenotypic diversity of monogenic hypertension will be discussed.

## Genetics of Monogenic Hypertension

**Familial hyperaldosteronism (FH)** is a rare autosomal dominant disorder of hypertension. It encompasses a group of conditions characterized by early-onset severe hypertension, hypokalemia, metabolic alkalosis, and a high plasma aldosterone/renin ratio (ARR>20). According to the underlying genetic defect, FH can be divided into four types, from I to IV. Diagnostically, it is hard to distinguish various subtypes of FH from sporadic primary aldosteronism (PA) just by clinical and biochemical observations, raising up the importance of genetic testing.

**FH-I** was firstly described by Sutherland et al. (12), representing about <1% of PA patients (13). For glucocorticoids have a significant positive effect on the disease, it was also named glucocorticoid remediable aldosteronism (GRA). In 1992, Lifton et al. (14) discovered the root cause of FH-I. Asymmetrical cross-over between the 11β-hydroxylase gene (*CYP11B1*) and the aldosterone synthase gene (*CYP11B2*) results in a chimeric gene, which possesses an adrenocorticotropic hormone (ACTH)-responsive promotor region at the 5' end of *CYP11B1* and the aldosterone synthase coding region at the 3' end of *CYP11B2*. The gold standard diagnosis is to confirm the chimeric gene by genetic sequencing. PA subjects combined with a PA-positive family history, early stroke, or early onset of the disease are recommended to take a genetic diagnosis for FH-I (15). Affected individuals are associated with a high incidence of the thoracoabdominal aneurysm and cerebrovascular complications (such as cerebral aneurysm, hemorrhagic stroke) (16, 17). In terms of treatment, low dose glucocorticoid is advised to inhibit the concentration of ACTH. Moreover, the key therapeutic target is normal smooth blood pressure, not the normalization of all biochemical parameters, which may lead to unnecessary side effects.

**FH-II**, first reported by Stowasser et al. (18), might be the most common form of FH. In 2018, Scholl et al. (19) demonstrated a gain of function mutation of the gene *CLCN2* in several affected subjects, which encodes voltage-gated chloride channel, ClC-2, that is expressed in adrenal glomerulosa. The mutation makes the glomerular cell membrane depolarize easily and activate voltage-gated calcium channels, thereby upregulating the expression of *CYP11B2* which encodes aldosterone synthase, the sole enzyme for aldosterone biosynthesis (20). Recently, a specific hypertensive mouse model with the missense mutation homologous to the *CLCN2* mutation related to the most frequent FH-II was reported by Schewe et al. (21) that further confirmed the role of ClC-2 in the regulation of aldosterone generation. Compared with FH-I, patients with FH-II are also associated with aldosterone-producing adenoma (APA) or bilateral adrenal hyperplasia (BAH). According to previous diagnostic criteria, when two or more family members suffer from PA, FH-II can be diagnosed after excluding the possibility of other FH forms (22). With the discovery of the new pathogenic gene variant, genetic testing may be a standard method for diagnosing FH-II. Different from FH-I, FH-II is unresponsive to glucocorticoids, but unilateral adrenalectomy combined with mineralocorticoid antagonists can relieve symptoms.

**FH-III** accounts for about 0.3% of PA (23) and is presumably caused by mutation of *KCNJ5* (24), which encodes a G protein-activated inward rectifying potassium channel, GIRK4. The mutation affects GIRK4 selectivity, leading to loss of potassium selectivity in adrenal cortical cells, enhancing sodium conductance, and an increased influx of sodium and depolarization of the membrane, finally resulting in elevated expression of *CYP11B2* (25). So far, six different germline mutations in *KCNJ5* gene have been identified, and the severity of clinical manifestation is variable (26). Severe hyperaldosteronism symptoms and BAH are typical features of FH-III patients. Adrenal computed tomography (CT) and adrenal venous sampling are necessary to indicate the disease, and genetic testing should be suggested for patients with PA. Bilateral adrenalectomy and mineralocorticoid antagonists usually dramatically alleviate symptoms.

**FH-IV** is a rare form of FH with an unclear prevalence worldwide that is caused by germline *CACNA1H* mutations (27). *CACNA1H* is abundantly expressed in the adrenal zona glomerulosa and encodes the α subunit of a T-type calcium channel (Cav3.2). Abnormal activation of Cav3.2 increases calcium influx, leading to abnormal aldosterone synthesis (28). Clinically, the symptoms of FH-IV are similar to other forms of FH, with low specificity. Gene testing is advised for young (≤10 years old) with hypertension and PA (29). There is currently no specific treatment for FH-IV. Based on the severity of a patient's condition, clinicians generally administer mineralocorticoid antagonists or perform adrenalectomy (27).

**Figure 2:**
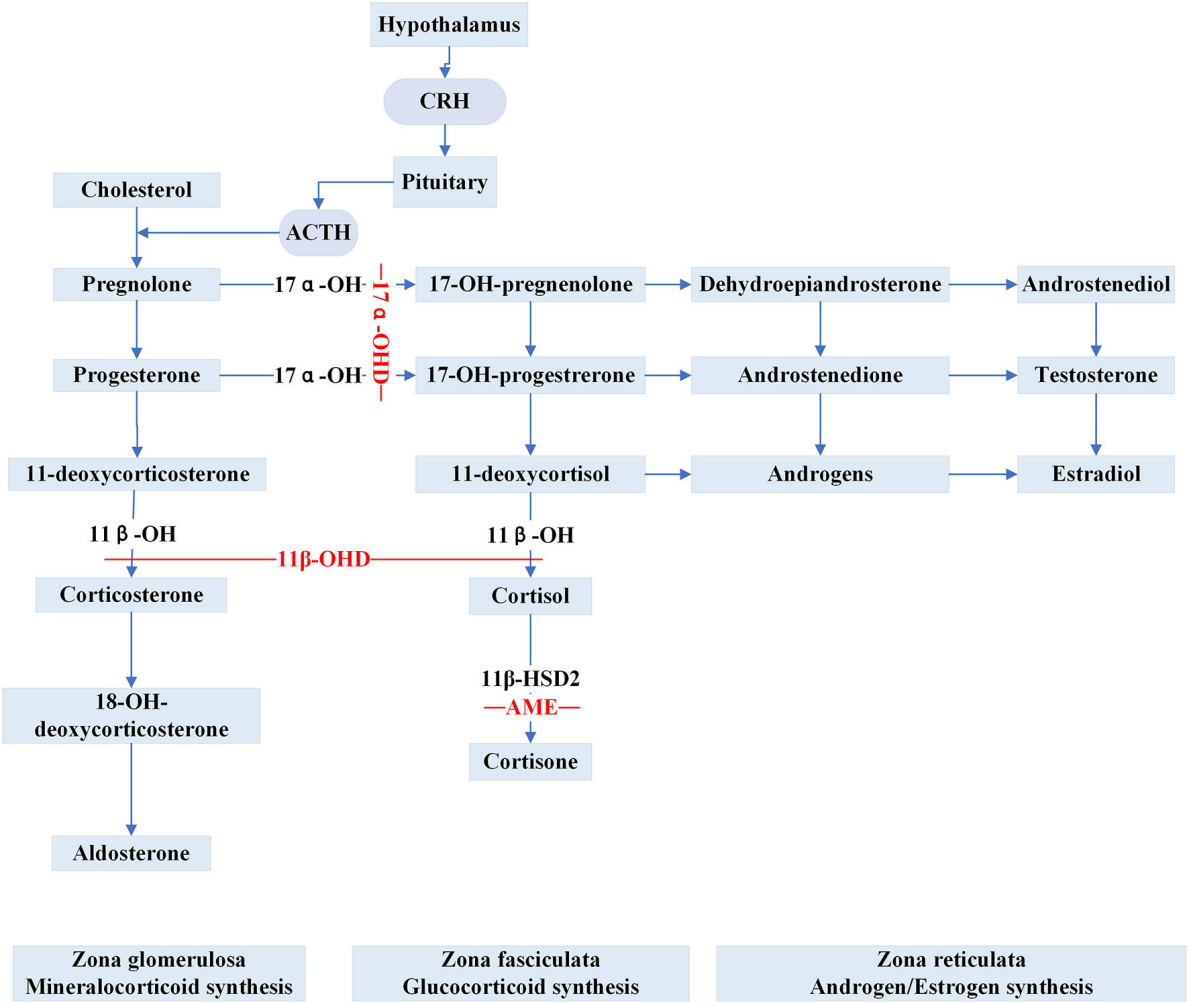
Steroid synthesis pathway and reduced activity of peripheral steroid hormone-metabolizing enzymes that catalyze the synthesis or inactivation of the corresponding hormones (11β-OHD, 17α-OHD, AME). 11β-OH, 11β-hydroxylase enzyme; 11β-OHD, 11β-hydroxylase deficiency; 17α-OH, 17α-hydroxylase enzyme; 17α-OHD, 17α-hydroxylase deficiency; 11β-HSD2, 11β-hydroxysteroid dehydrogenase type 2; AME, apparent mineralocorticoid excess.

**Apparent Mineralocorticoid Excess (AME)** is inherited as an autosomal recessive trait caused by inactivating *HSD11B2* variants. The gene is located at cytogenetic locus 16q and is responsible for the synthesis of 11β-hydroxysteroid dehydrogenase type 2 (11β-HSD2) (30). 11β-HSD2 is a member of the short-chain dehydrogenase/reductase family and is richly expressed in the salivary gland, skin, colon, placenta, thymus, vascular vessel, kidney and brain (31, 32). In renal epithelial tissue, 11β-HSD2 is principally co-localized with the mineralocorticoid receptors (MRs) in aldosterone-sensitive distal nephron (ASDN) (33). The MRs share a similar affinity for aldosterone and cortisol without selective characteristics and the circulating concentration of cortisol is 100–1000 times higher than aldosterone (33). Physiologically, the enzyme 11β-HSD2 catalyzes the metabolic conversion of cortisol to cortisone and corticosterone to 18-OH-deoxycorticosterone, respectively and thereby prevents the MRs from cortisol over-saturation (Figure 2). However, because of the mutated gene, the metabolic impairment of cortisol results in cortisol-mediated overstimulation of the MRs. It is worth noting that the clinical phenotype of AME is variable, and based on varying degrees of enzyme deficiency, AME can be divided into two types: a severe phenotype (AME-I) and a mild phenotype (AME-II). Patients with homozygous mutations display low birth weight, developmental retardation, unrelenting salt-sensitive hypertension in childhood. Nevertheless, those patients with heterozygous mutations usually show a mild or moderate phenotype, such as late-onset slight hypertension or staying normotensive, barely with electrolyte disturbances (34, 35). Previously, a high ratio of urinary cortisol to cortisone (F/E) was considered a basis for AME diagnosis. Recent studies (36, 37) show that serum F/E and urinary exosomes miRNA levels are more sensitive for the prediction of AME-II. In addition, it is necessary to distinguish the disease from those with acquired suppression of the activity of *HSD11B2* by the ingestion of excessive liquorice or carbenoxolone (38). Treatments including MR antagonist spironolactone, dexamethasone can effectively alleviate AME.

**Congenital adrenal hyperplasia (CAH)** is a class of steroidogenic disorders transmitted in an autosomal recessive fashion. It is caused by variants in genes encoding enzymes involved in adrenal steroid synthesis. 11β-hydroxylase deficiency (11β-OHD) and 17α-hydroxylase deficiency (17α-OHD) are two forms of CAH typically showing early-onset hypertension combined with hypokalemia caused by the accumulation of ACTH and mineralocorticoids. Figure 2 shows detailed pathways for the synthesis of steroids.

**11β-OHD** is the second major subtype of CAH, accounting for 0.2–8% of all CAH cases while 21-hydroxysteroid deficiency is the most common subtype of CAH (39, 40). Mutation in *CYP11B1*, encoding steroid 11β-hydroxylase (11β-OH), results in a series of pathophysiological changes, and the mutation has a significant racial specificity (41). Deficiency of 11β-OH leads to impaired conversion of 11-deoxycortisol and 11-deoxycorticosterone to cortisol and corticosterone, respectively, resulting in an elevated ACTH level and superfluous adrenal androgens. Typically, apart from mineralocorticoid excess, patients may display hyperandrogenemia, which leads to masculinization of genitalia in females, pseudoprecocious puberty in males, and premature bone maturation in both. Curiously, the degree of excessive MR activation is not strongly correlated with the extent of virilization. Some severely virilized women have normal blood pressure, while mild individuals may experience resistant hypertension or even fatal cardiovascular accidents (42). Because of the variability of clinical manifestations, the possibility of 11β-OHD should be considered when encountering hypertensive patients with hyperandrogenemia and hyperadrenocorticism (41). The raised levels of 11-deoxycortisol, 11-deoxycorticosterone, and androgen are robust clues for the biochemical diagnosis of suspected 11β-OHD patients. Appropriate glucocorticoid supplementation is helpful in improving hypertension, hypokalemia and end-organ damage (43). Some patients with refractory hypertension or severe hyperandrogenism that is unresponsive to glucocorticoid therapy are recommended to undergo bilateral adrenalectomy. If necessary, surgical correction for patients with genital deformities is feasible according to the patients' will. Moreover, aromatase inhibitor therapy may be useful to improve the height prognosis of 11β-OHD patients, especially for those with very late bone ages (44).

**17α-OHD** is a syndrome caused by the mutation in *CYP17A1*. The function of 17α-hydroxylase enzyme (17α-OH) is to catalyze the conversion of pregnenolone/progesterone to 17-OH-prenenolone/17-OH-progesterone, respectively. Defective 17α-OH causes the insufficient secretion of cortisol, gonadal steroids, adrenal androgens, and because of a feedback increase in ACTH, the production of excess mineralocorticoids (45). Classical characteristics are sexual infantilism (secondary sexual sign hypoplasia, primary amenorrhea), and delayed puberty. Recently, Wu et al. (46) reported reduced bone density and adrenal masses in affected cases, but the underlying pathogenesis is not yet clear. Physical and biochemical tests are useful for detecting disease, while genetic sequencing is a standard way to confirm a diagnosis. Routine treatment of 17α-OHD includes MR antagonists, supplement of sex steroids and glucocorticoid; if the defective karyotype is 46, XY, prophylactic gonadectomy is also executed to prevent the genesis of gonadal tumors (47, 48). Clinicians should also pay close attention to the bone mineral density of patients with prolonged glucocorticoid therapy to prevent the occurrence of adverse events such as fractures (49).

**Glucocorticoid resistance (GCCR)** is a hereditary monogenic disorder transmitted in an autosomal dominant pattern, caused by variants in *NR3C1*, which encodes the glucocorticoid receptor (GR). The defective mutated gene leads to glucocorticoid resistance, which may be due to impaired GR binding affinity, reduced GR number, or impaired GR signal transduction function (50–52). The inactivation of GR results in a feedback activation of the hypothalamic-pituitary-adrenal (HPA) axis with the augmentation of ACTH, steroids, such as cortisol and androgens. Consequently, residual cortisol and mineralocorticoid precursor substances (deoxycortisol and corticosterone) are associated with resistant hypertension and hypokalemia. The typical features are characterized by a significant increase in plasma cortisol and ACTH levels, virilization in females and pseudoprecocious in males but without evidence of Cushing syndrome (53). Also, fatigue and anxiety are common characteristics of GCCR (54). High serum cortisol and urinary free cortisol are sensitive biomarkers, and their levels do not decrease after adrenal suppression by dexamethasone leading to the suspicion of GCCR. Low dose dexamethasone can be considered to suppress the secretion of ACTH, subsequently reducing the over-production of mineralocorticoids and androgens. To antagonize the androgen effect, clinicians sometimes administer aldosterone antagonists (53). When facing complex complications like cerebral infarction caused by severe long-term hypertension, high doses of dexamethasone are suitable (55).

**Geller syndrome**, also known as pregnancy-exacerbated hypertension, is a rare autosomal dominant disorder resulting from a gain-of-function mutation in the MR gene *NR3C2*, and is characterized by its constitutive activation (56). Changes in the selectivity and affinity of mutant MR for its ligand lead to their abnormal activation not only by aldosterone but also some steroids cortisone and progesterone (57). The disease typically presents at an early age and shows excess mineralocorticoid symptoms. The level of progesterone is significantly upregulated during pregnancy; therefore, pregnant women with the mutation display more severe symptoms (58). Definitive diagnosis can be achieved by genetic sequencing. The MR antagonist, spironolactone, is absolutely contraindicated because it is an agonist for mutant MR. For pregnant women with exacerbated hypertension, termination of pregnancy is advised to improve symptoms efficiently.

**PCC** refers to tumors originating from adrenal chromaffin cells with an estimated incidence of 3–8 cases per million people per year (59), and the ones arising from extra-adrenal chromaffin tissues are called paraganglioma (PGL). Germline mutations exist in more than 40% of patients with PCC or PGL, and it is mainly inherited in an autosomal dominant manner (60). Hereditary PCC can occur solitarily or as local manifestations of some genetic tumor syndromes, such as multiple endocrine neoplasia, neurofibromatosis, von Hippel-Lindau (61). Patients carrying susceptible genes are with the great possibility of developing PCC/PGL at a young age (62). The identification of susceptibility genes has been a research hotspot recently, which facilitates the diagnosis of patients in asymptomatic period (63). To date, the following tumor susceptibility genes have been reported: *KIF1B, SDHB, TMEM127, VHL, GDNF, RET, SDHD, MAX, SDHC, SDHA, SDHAF2, NF1*, with further novel mutations continually being discovered (64, 65). Continuous or paroxysmal hypertension, headaches, palpitations, diaphoresis, pallor are typical symptoms because of high concentrations of catecholamines. Genetic testing should be considered in all patients with PCC/PGL, particularly for those with a positive family history, or single and multifocal metastasis (64, 66, 67). Plasma free metanephrines (MNs) or all-day urinary free MNs are sensitive markers for PCC while spot urinary free MNs/creatinine is a powerful biomarker for PGL (68, 69). Imaging modalities such as CT, magnetic resonance imaging (MRI), positron emission tomography/CT (PET/CT) are beneficial for tumor localization. After a definite diagnosis, functional PCC is often removed surgically with α-adrenergic antagonists taken during the perioperative period. Annual biochemical follow-up of patients after surgery should last at least 10 years, longer for high-risk patients (64).

**Liddle syndrome (LS)** is an autosomal dominant disorder caused by gain-of-function mutations in the genes that encode the epithelial sodium channel (ENaC) subunits. Germline mutations in *SCNN1A, SCNN1B, SCNN1G*, encoding α, β, and γ subunits, respectively, lead to sodium reabsorption and blood volume expansion by affecting specific characteristics of the ENaC (70). In comparison, mutations in *SCNN1B* and *SCNN1G* are the most frequent forms of LS (8). In 2017, Salih et al. (71) reported a Caucasian family with LS and identified a new heterozygous missense mutation in *SCNN1A* through exome sequencing. Definitive diagnosis mainly depends on genetic screening. Early diagnosis and therapy are necessary for LS can cause sudden death, stroke, and end-stage renal failure (8). However, the prevalence of LS in the hypertensive population is still unclear. Two studies have shown the prevalence of LS among Chinese young hypertensive patients to be 1.52% and 0.91% (72, 73). Nevertheless, the discovery of new pathogenic genes and variable penetrance indicates that the prevalence of LS should be much higher than currently estimated. LS patients show remarkable response to the ENaC blocker, amiloride, and the potassium-sparing diuretic, triamterene. A salt-limited diet is also recommended on the basis of medication (74). The long-term prognosis of LS is not clear and requires further investigation.

## Pathophysiology of Monogenic Hypertension

Plasma volume expansion and catecholamines/sympathetic excess are two distinct mechanisms that contribute to hypertension, the former being the more common cause. ENaC channels are abundantly distributed in the apical portion of epithelial cells of the connecting duct and the distal convoluted tubule. ENaC controls the rate-limiting step of sodium reabsorption from the lumen into the epithelial cells in the final pathway of volume expansion. There is a highly conserved proline-rich region named the PY motif in all carboxyl-terminal of three subunits. The ubiquitin ligase, NEDD4-2, can bind to the PY motif and initiate the ubiquitination of ENaC and endocytotic degradation (75). Mutations in ENaC subunit genes can directly increase the density and the open probability of channels resulting in sodium retention (LS).

In addition, an important factor influencing the expression of ENaC channels is excessive mineralocorticoid effects, which can be divided into three pathways: (1) abnormal affinity of steroid hormone receptors resulting from MR and GR genes variants (Geller Syndrome and GCCR); (2) reduced activity of peripheral steroid hormone-metabolizing enzymes that catalyze the synthesis or inactivation of corresponding hormones (AME, 11β-OHD, 17α-OHD); (3) excessive aldosterone synthesis (FH-I, II, III, IV). The over-bound of mineralocorticoid/MR complex up-regulates the expression of serum and glucocorticoid-inducible kinase 1 (SGK1), thus regulating the activity of ENaC in various ways. First, SGK1 directly phosphorylates NEDD4-2 by binding to its amino acid residues and reduces the ubiquitination of ENaC channels. Second, SGK1 can promote the combination of NEDD4-2 and 14-3-3 protein, leading to the conformational change of NEDD4-2, which diminishes the NEDD4-2/ ENaC interaction (76). Moreover, SGK1 could enhance the electrophysiological function of ENaC through directly phosphorylating or influencing the transcription process of its α subunit (77).

Catecholamines/sympathetic excess is another mechanism of monogenic hypertension that occurs in PCC/PGL. Various clusters' genetic mutations which involve activation of hypoxia-angiogenic pathways or RAS and kinase signaling pathways lead to the development of catecholamine-producing neuroendocrine neoplasms (64). Activation of the sympathetic nervous system mediates the contraction of the renal vasculature, hence stimulating the secondary secretion of renin and aldosterone which, in turn, increases the reabsorption of sodium and water (78).

## Pathophysiology of Hypokalemia

Increased potassium excretion in the urine and intracellular shift are two common causes of hypokalemia, a typical electrolyte disturbance of monogenic hypertension. The kidney plays a vital role in maintaining potassium balance in the body. Under normal physiological conditions, 90% of excreted potassium is passed in the urine with only 10% via the digestive tract. When 24-h urinary potassium is >15 mEq, inappropriate renal potassium loss is indicated (15). Differential transmembrane concentrations of potassium and potential gradients drive tubule potassium secretion via the reabsorption of sodium through ENaC located in the distal convoluted tubule. The activity of ENaC can be elevated through mineralocorticoid effects by increasing the number of channels and the channel opening time, which is responsible for partial forms of monogenic hypertension combined with hypokalemia.

Hyperactive ENaC and excess mineralocorticoid effect are associated with the elevated secretion of potassium. Physiologically, sodium ions in the lumen enter the cell through ENaC along the electrochemical gradient and are pumped across the basolateral membrane through the Na^+^/K^+^-ATPase, which drives the transport of potassium into the cell, followed by the secretion into urine through different potassium channels (79). Due to hyperactive ENaC, the increase of sodium reabsorption results in a luminal negative potential gradient, stimulating the elevated potassium secretion to maintain electrochemical equilibrium. Similarly, excess mineralocorticoid effects magnify the activity of ENaC, followed by too much potassium loss. The renal outer medullary K^+^ channel located in the distal tubule is one of the key transport portions of potassium (80). Maxi-K channel also plays a vital role in secreting potassium, whose function could be activated by increased luminal flow rate and aldosterone (81, 82).

The vast majority of potassium (98%) is present in intracellular fluid. The maintenance of potassium distribution between intracellular and extracellular fluids, referred to as the internal potassium balance, is also an intrinsic mechanism for potassium homeostasis. Insulin and catecholamines are the two most important hormones that affect the internal potassium balance by moving potassium into cells (82). The high levels of catecholamines released by PCC can increase the activity of Na^+^/K^+^-ATPase by blocking the α receptors or activating β_2_ receptors, thus facilitating the migration of potassium into cells. In a nutshell, the extent of sodium reabsorption in the ASDN, the flow rate of fluid in the distal convoluted tubule, acid-base disorders, the state of α/ β_2_ receptors, and the concentration of aldosterone and arginine vasopressin are all key factors in determining the degree of potassium secretion.

## Phenotypic Heterogeneity

The clinical phenotypes and genotypes of monogenic hypertension show considerable heterogeneity, which may hamper the diagnosis of the disease. With the same genetic mutation, patients may display various degrees of clinical manifestation, ranging from milder symptoms encompassing normotension or normokalemic to severe, life-threatening conditions (20, 46, 70, 83, 84). Genetic interaction, epigenetic modifier and non-genetic factors, such as age, environmental and nutritional factors, are closely associated with the variable phenotype of monogenic hypertension.

## Genotype-Phenotype Correlation

Firstly, some mutations which result in partial activity defects of relevant enzymes are possibly responsible for some mild phenotypes, which may be misdiagnosed easily (38, 85). For instance, patients with the mutations in exon 3 of gene *CYP11B1* tend to present as a non-classic phenotype, similar to polycystic ovary syndrome (41). Second, the overlap of germline and somatic mutations may be one of the causes of phenotypic variability. Lin et al. (86) first reported two GRA affected siblings, who also had a somatic *KCNJ5* mutation, showing the rare phenotype of adrenal adenoma, hypertension and lower blood potassium level compared with most GRA patients. In addition, mutations in different regions of ClC-2 may affect channel activity to varying degrees, partly explaining the phenotypic heterogeneity of FH-II (87).

## Epigenetic Modification

Epigenetic modification plays a critical role in explaining the phenotypic heterogeneity of monogenic hypertension beyond genetic defects. It refers to a reversible change in gene function but without altering the DNA sequence in the nucleus. DNA methylation is one of the first modification pathways to be discovered. High methylation levels of the *HSD11B2* promoter are associated with increased blood pressure and is one of the factors influencing the onset of hypertension (88). Multiple lines of evidence show that the higher the degree of *HSD11B2* promoter methylation, the lower the activity of 11β-HSD2, which possibly explains the inconsistent phenotype of hypertension among AME patients (9, 89). Furthermore, the role of non-coding microRNAs and histone modification in regulating blood pressure has been reviewed by Burrello et al. (90), but the mechanisms of these process that affect the phenotype of monogenic hypertension remain to be identified.

## Non-Genetic Factors

Age along with environmental and nutritional factors, including obesity, diabetes, daily sodium and potassium intake, may also have an effect on the disease phenotype (11, 91). Tapolyai et al. (92) reported the phenomenon of elderly patients demonstrating Liddle's-like syndrome but with the negative family history. Several elderly patients presenting with Liddle syndrome phenotype also have been discovered and identified without genetic mutation, rasing the hypothesis that there is a correlation between age or medication and ENaC channel activity (93, 94). Several studies (95, 96) have confirmed that the level of cortisol and the ratio of F/E decrease with age and suggested the activity of 11β-HSD2 gradually reduces with age, which may relate to variable clinical phenotype of AME. The the exact mechanism between sodium, potassium intake levels and the diverse phenotype of monogenic hypertension deserves further study.

## Conclusions

Table 1 summarizes the basic characteristics of different monogenic forms of hypertension combined with hypokalemia. Although the epidemiology of monogenic hypertension is not completely clear and requires further study, we suggest that monogenic hypertension is not an uncommon cause of secondary hypertension, especially for those with simultaneous electrolyte disturbances. We do require useful strategies to identify phenotypic heterogeneity to avoid severe complications of monogenic hypertension. Genetic testing, which is precise and efficient, is beneficial for the early identification of patients, for targeted therapy and better management of affected subjects, particularly for those with atypical phenotypes, thus improving the prognosis of the disease. Overall, there is great optimism that the morbidity and mortality of monogenic hypertension will decrease further with the advancement of genetically driven individualized treatment.

**Table 1:** Basic characteristics of different monogenic forms of hypertension with hypokalemia.

**Forms**		**OMIM phenotype number**	**Genetic mutation**	**Location**	**Inheritance pattern**	**Encoded protein**	**Biochemical results**		**Clinical manifestations**	**Confirmatory test**	**Management**
							**PRA**	**PAC**			
Familial hyperaldosteronism	Type-I	#103900	*CYP11B1/CYP11B2* hybrid gene	8q24.3	AD	ADS	↓	↑	early-onset hypertension, metabolic alkalosis, remediable with glucocorticoid	ARR, remediable with glucocorticoid, gene testing	glucocorticoid supplementation
	Type-II	#605635	*CLCN2*	3q27.1	AD	ClC-2	↓	↑	refractory hypertension, APA, BAH, no response to glucocorticoid	imaging modalities, gene testing	adrenalectomy, MRA
	Type-III	#613677	*KCNJ5*	11q24.3	AD	GIRK4	↓	↑	severe hypertension, BAH, early damage to target organs	imaging modalities, adrenal venous sampling, gene testing	bilateral adrenalectomy, MRA
	Type-IV	#617027	*CACNA1H*	16p13.3	AD	Cav3.2	↓	↑	early-onset hypertension, APA	gene testing	from MRA to adrenalectomy
Apparent Mineralocorticoid Excess		#218030	*HSD11B2*	16q22.1	AR	11β-HSD2	↓	↓	from severe (low birth weight, developmental retardation) to milder (mild hypertension and rare electrolyte abnormalities) phenotypes	urinary or serum F/E, gene testing	MRA, dexamethasone, potassium-sparing diuretic
Congenital adrenal hyperplasia	11β-OHD	#202010	*CYP11B1*	8q24.3	AR	11β-OH	↓	↓	hyperandrogenemia, severe virilization, short stature	levels of S, DOC and androgen, gene testing	glucocorticoids supplementation, bilateral adrenalectomy, MRA
	17α-OHD	#202110	*CYP17A1*	10q24.32	AR	17α-OH	↓	↓	secondary sexual sign hypoplasia, primary amenorrhea, delayed puberty	levels of S, DOC and androgen, gene testing	MRA, supplement of sex steroids and glucocorticoid, prophylactic gonadectomy
Geller syndrome		#605115	*NR3C2*	4q31.23	AD	MR	↓	↓	pregnancy-exacerbated hypertension, early age refractory hypertension	gene testing	termination of pregnancy, no MRA
Glucocorticoid resistance		#615962	*NR3C1*	5q31.3	AD	GR	↓	↓	hypoglycemia, hypercortisolism, hyperandrogenism	serum/urinary cortisol, gene testing	dexamethasone administration, MRA
Familial pheochromocytoma/paraganglioma		#171300	*KIF1B*	1p36.22	AD	Kinesin-like protein KIF1B	↑	↑	continuous or paroxysmal hypertension, headaches, palpitations, diaphoresis, pallor	levels of CA, imaging modalities, gene testing	surgery, α-adrenergic antagonists
		#171300	*SDHB*	1p36.13		Succinate dehydrogenase iron-sulfur subunit					
		#171300	*TMEM127*	2q11.2		Transmembrane protein 127					
		#171300	*VHL*	3p25.3		pVHL					
		#171300	*GDNF*	5p13.2		Glial cell-line derived neurotrophic factor					
		#171300	*RET*	10q11.21		Proto-oncogene tyrosine-protein kinase receptor Ret					
		#171300	*SDHD*	11q23.1		Succinate dehydrogenase cytochrome b small subunit					
		#171300	*MAX*	14q23.3		Protein max					
		#605373	*SDHC*	1q23.3		Succinate dehydrogenase cytochrome b560 subunit					
		#614165	*SDHA*	5p15.33		Succinate dehydrogenase flavoprotein subunit					
		#601650	*SDHAF2*	11q12.2		Succinate dehydrogenase assembly factor 2					
		#162200	*NF1*	17q11.2		Neurofibromin					
Liddle syndrome		#177200	*SCNN1B*	16p12.2	AD	ENaC	↓	↓	early-onset hypertension, metabolic alkalosis, hypokalemia	gene testing	amiloride, triamterene and a salt-limited diet
		#618114	*SCNN1G*	16p12.2							
		#618126	*SCNN1A*	12p13.31							

*PRA, plasma renin activity; PAC, plasma aldosterone concentration; AD, autosomal dominant; AR, autosomal recessive; ↑, high; ↓, low; ADS, aldosterone; ARR, plasma aldosterone/rennin ratio; APA, aldosterone producing adenoma; BAH, bilateral adrenal hyperplasia; MRA, mineralocorticoid receptor antagonist; F/E, cortisol to cortisone ratio; 11β-HSD2,11β-hydroxysteroid dehydrogenase type 2 enzyme; 11β-OHD,11β-hydroxylase deficiency; 11β-OH, 11β-hydroxylase enzyme; S, 11-deoxycortisol; DOC, 11-deoxycorticosterone; 17α-OHD,17α-hydroxylase deficiency; 17α-OH,17α-hydroxylase enzyme; MR, mineralocorticoid receptor; GR, glucocorticoid receptor; CA, catecholamines; ENaC, epithelial sodium channel*.

## Author Contributions

All authors are responsible for the literature review, drafting and revision of the manuscript, and approved the final version of the manuscript.

## Conflict of Interest

The authors declare that the research was conducted in the absence of any commercial or financial relationships that could be construed as a potential conflict of interest.
